# Supplementary material for: Loss of DNA Polymerase β Delays Atherosclerosis in ApoE−/− Mice Due to Inhibition of Vascular Smooth Muscle Cell Migration
Source: Int J Mol Sci. 2024 Nov 2;25(21):11778. doi: 10.3390/ijms252111778 (PMC11547094; doi:10.3390/ijms252111778)
Supplement: Supplementary file 1 [file ijms-25-11778-s001.zip › Table S1.pdf]

Table S1. Antibody information.

**Name**	**Brand**	**Number**	**Antibody stock solution: PBS**
β-tubulin	Abclonal	AC015	1:1000
Pol β	Abcam	ab175197	1:1000
Periostin	Abmart	M032144	1:3500
YY1	Abcam	ab109237	1:1500
TGF-β1	Abclonal	A2124	1:4000
GAPDH	Abclonal	Ac036	1:1000
E-cadherin	Abclonal	A20798	1:1000
N-cadherin	Proteintech	22018-1-AP	1:1000
Vimentin	Abcam	ab92547	1:500
α-SMA	Abclonal	A7248	1:1000
