# Supplementary material for: Loss of DNA Polymerase β Delays Atherosclerosis in ApoE−/− Mice Due to Inhibition of Vascular Smooth Muscle Cell Migration
Source: Int J Mol Sci. 2024 Nov 2;25(21):11778. doi: 10.3390/ijms252111778 (PMC11547094; doi:10.3390/ijms252111778)
Supplement: Supplementary file 1 [file ijms-25-11778-s001.zip › Table S2.pdf]

Table S2. Primer sequences.

Gene	Primer sequence (5′ → 3′)	Sequence	length	Purification method	Manufacturer
Homo-Pol β	Forward Primer	5’-CCGCAGGAGACTCTCAACG-3’	19	HPLC	TSINGKE
	Reverse Primer	5’-GTACTTGTGGATAGCTTGGCTC-3’	22	HPLC	TSINGKE
Mus-Pol β	Forward Primer	5’-GCGTACAGAAAAGCGGCATC-3’	20	HPLC	TSINGKE
	Reverse Primer	5’-TGTTCCTACTCCTGGCAGTTTC-3’	22	HPLC	TSINGKE
Homo-β-Actin	Forward Primer	5’- ACATCCGCAAAGACCTGTAC-3’	20	HPLC	TSINGKE
	Reverse Primer	5’- CAGGGCAGTGATCTCCTTCT-3’	20	HPLC	TSINGKE
Homo-COL1α1	Forward Primer	5’-GAGGGCCAAGACGAAGACATC-3’	21	HPLC	TSINGKE
	Reverse Primer	5’-CAGATCACGTCATCGCACAAC-3’	21	HPLC	TSINGKE
Mus-COL1α1	Forward Primer	5’-GCTCCTCTTAGGGGCCACT-3’	19	HPLC	TSINGKE
	Reverse Primer	5’-ATTGGGGACCCTTAGGCCAT-3’	20	HPLC	TSINGKE
Homo-COL3α1	Forward Primer	5’-AGATGTACCGTTGGTGCCTG-3’	20	HPLC	TSINGKE
	Reverse Primer	5’-CGTCCTTTGATTCTCGGCCT-3’	20	HPLC	TSINGKE
Mus-COL3α1	Forward Primer	5’-ACTGCAAAAACAGTATTGCCT-3’	21	HPLC	TSINGKE
	Reverse Primer	5’-TCTATGATGGGTAGTCTCATTGCCT-3’	25	HPLC	TSINGKE
Homo- COL5α1	Forward Primer	5’-TACCCTGCGTTCTGCATTTCC-3’	21	HPLC	TSINGKE
	Reverse Primer	5’-GCTCGTTGTAGATGGAGACCA-3’	21	HPLC	TSINGKE
Homo-COL6α1	Forward Primer	5’-GAGCTGGTCAAGTTCGAGCC-3’	20	HPLC	TSINGKE
	Reverse Primer	5’-CCACTGCAGGCTCTTGATGG-3’	20	HPLC	TSINGKE
Mus-COL6α1	Forward Primer	5’-GATGAGGGTGAAGTGGGAGA-3’	20	HPLC	TSINGKE
	Reverse Primer	5’-CAGCACGAAGAGGATGTCAA-3’	20	HPLC	TSINGKE
Homo-COL6α2	Forward Primer	5’- TGCTCCGTGCTCCTGCTCTG-3’	20	HPLC	TSINGKE

	Reverse Primer	5’- TGTGCCTGTTTCTGACTTGG-3’	20	HPLC	TSINGKE
Mus-COL6α2	Forward Primer	5’- ATGTGAGGGAGACCTGTGGA-3’	20	HPLC	TSINGKE
	Reverse Primer	5’- TGTGCCTGTTTCTGACTTGG-3’	20	HPLC	TSINGKE
Homo-YY1	Forward Primer	5’- AAGAGCGGCAAGAAGAGTTAC-3’	21	HPLC	TSINGKE
	Reverse Primer	5’- CAACCACTGTCTCATGGTCAATA-3’	23	HPLC	TSINGKE
Homo-TGFβ1	Forward Primer	5’- TGCACATTGCCTGTTCTGCT-3’	20	HPLC	TSINGKE
	Reverse Primer	5’- TGCATCTTGGTTGGCTGCAT-3’	20	HPLC	TSINGKE
Homo-MMP2	Forward Primer	5’- GTGAAGTATGGGAACGCCG-3’	19	HPLC	TSINGKE
	Reverse Primer	5’- GCCGTACTTGCCATCCTTCT-3’	20	HPLC	TSINGKE
Homo-MMP3	Forward Primer	5’- ACATGGAGA CTTTGTCCCT TTTG-3’	23	HPLC	TSINGKE
	Reverse Primer	5’- TTGGCTGAGTGGTAGAGTCCC-3’	21	HPLC	TSINGKE
Homo-MMP9	Forward Primer	5’- AGACCTGGGCAGATTCCAAAC-3’	21	HPLC	TSINGKE
	Reverse Primer	5’- CGGCAAGTCTTCCGAGTAGT-3’	20	HPLC	TSINGKE
Homo-MMP13	Forward Primer	5’- CCAGACTTCACGATGGCATTG-3’	21	HPLC	TSINGKE
	Reverse Primer	5’- GGCATCTCCTCCATAATTTGGC-3’	22	HPLC	TSINGKE
Homo-ApoE	Forward Primer	5’-CGCTTTTGGGATTACCTGCG-3’	20	HPLC	TSINGKE
	Reverse Primer	5’-GGGGTCAGTTGTTCCTCCAG-3’	20	HPLC	TSINGKE
Mus-ApoE	Forward Primer	5’-ACTTAGCCGGGAAGAAAGAGGA-3’	22	HPLC	TSINGKE
	Reverse Primer	5’-CCACTCGAGCTGATCTGTCAC-3’	21	HPLC	TSINGKE
Homo-Twist1	Forward Primer	5’- GTCCGCAGTCTTACGAGGAG-3’	20	HPLC	TSINGKE
	Reverse Primer	5’- GCTTGAGGGTCTGAATCTTGCT-3’	22	HPLC	TSINGKE
Homo-POSTN	Forward Primer	5’-GCTATTCTGACGCCTCAAAACT-3’	22	HPLC	TSINGKE
	Reverse Primer	5’-AGCCTCATTACTCGGTGCAAA-3’	21	HPLC	TSINGKE
Homo-sh Pol β	Sense	5’-GGAGCTGAAGCTAAGAAATTG-3’	21	HPLC	TSINGKE
Homo-si POSTN	Sense	5’-CGCAAATGGGCGGTAGGCGTG-3’	21	HPLC	TSINGKE
